# Situated Precision Healthcare in the Smart Medical Home: Bringing NASA’s Research Strategy down to Earth

**DOI:** 10.20900/agmr20200017

**Published:** 2020-06-29

**Authors:** Gary Riccio, Rhoda Au, Richard van Emmerik, Mohammed Eslami

**Affiliations:** 1 Nascent Science Technology LLC, Boston, MA 02108, USA; 2 Department of Neurology and Alzheimer’s Disease Center, Boston University School of Medicine, Boston, MA 02118, USA; 3 Framingham Heart Study, National Heart, Lung, and Blood Institute, Boston, MA 01702, USA; 4 Department of Epidemiology, Boston University School of Public Health, Boston, MA 02118, USA; 5 Department of Anatomy Neurobiology, Boston University School of Medicine, Boston, MA 02118, USA; 6 School of Public Health and Health Sciences, University of Massachusetts, Amherst, MA 01003, USA; 7 Center for Personalized Health Monitoring, University of Massachusetts, Amherst, MA 01003, USA; 8 Netrias LLC, Cambridge, MA 02142, USA

**Keywords:** aging, digital biomarkers, systems medicine, precision health, isolation, confinement

## Abstract

This special issue is ambitious in that it calls for strategic transformation in research on Alzheimer’s Disease (AD) and related dementias, including innovation in both research design and value delivery, through lifestyle interventions that implicitly relate to a much broader range of comorbidities and diseases of aging. One response to this challenge is to venture beyond the boundaries of research that supports the healthcare industry. Toward this end, we introduce opportunities for research translation and knowledge transfer from NASA to the healthcare industry. Our intent is to show how NASA’s approach to research can guide innovation for a smart medical home, most notably for AD and other diseases of aging. The article is organized in four major sections: (a) aggregating fragmented research communities; (b) lifestyle interventions in the medical home; (c) multiscale computational modeling and analysis; and (d) lifespan approach to precision brain health. We provide novel motivations and transformative paths to a diversity of specific lines of research, across communities, that would be difficult to discover in common methods of networking within research communities and even through sophisticated bibliographic methods. We thus reveal how knowledge transfer between the public and private sector can stimulate development of broader scientific communities and achieve a more coherent strategic approach to integration and development of a diversity of capabilities including but not limited to technology.

## AGGREGATING FRAGMENTED RESEARCH COMMUNITIES

### A Vignette about Aging in Place

Mary is 91 years old and has lived alone at home for many years since her husband died. She only recently, at the age of 89, stopped working as a nurse, as she would say “helping the old people”. She drives to various places where she does volunteer work as well as in conducting quotidian activities such as going to the grocery store, drug store and post office. She also has regular visitors at her home, both friends and family who know her well. Her son John lives a few miles away in the same town and visits every day for a brief period of time. Her daughter Jane visits for extended periods of time most weekends and often stays overnight. Her other children, Benjamin and Eduardo, live far away and maintain contact mostly by phone. She strives to be part of the lives of her grandchildren and great grandchildren, who live far away, by learning and using modern technology as well as the telephone that is instrumental in her quality of life.

John likes to keep Mary active but, lately, he noticed that she has been a bit lethargic and, according to her friends in town, has withdrawn a bit from social activity. She seems to get confused from time to time. The family wonders if it is their imagination. Is it something related to aging, or is Mary just taking on too much? They are aware, of course, that aging brings decline in both cognitive and motor function, but they are unaware of relatively recent research that provides evidence for a causal connection and, specifically, an increased risk of falling [1,2]. Mary tells John that it probably is because Mary hasn’t been sleeping well. Jane has noticed that Mary has a tendency to become dehydrated. Sometimes she forgets to eat and drink. John and Jane share this information with Ben and Ed. All four of them begin calling Mary more often to get reports about her daily activities including sleep and nutrition. Mary slowly gets a bit more active, and she is sleeping and eating more reliably. She seems more alert, most of the time.

Mary wakes up one day filled with energy and enthusiasm. Looking forward to a lunch date with a friend, and with a renewed bounce in her step, she begins to descend the stairs from her bedroom while carrying a few items to throw in the laundry. The phone rings. She becomes excited, waiting to hear the audible caller identification. Her posture stiffens a bit in the excitement, and she simultaneously turns her head toward the side of the stairs beyond which the phone is located. She loses her balance, reaches for the banister, but she is unable to grab it as she often has in the past when feeling the need for more support. She lands face first on the tile floor at the bottom of the stairs, and she lies in a pool of blood. Fortunately, John’s wife Jennifer almost immediately comes into the room, sees the horror, and calls 911, literally saving Mary’s life.

After several days in intensive care and weeks in a rehabilitation facility, Mary begins recovery at home. While her physical injuries are slowly abating, her cognitive symptoms seem to have worsened. John, who is a contractor, converts Mary’s two-story home into a place where she can do everything on the main floor. Ed, a health and performance researcher, installs internet cameras in the home as well as voice actuated lighting and entertainment with the help of his brother John. Because of his project management experience, John realizes that having the resources in place is only part of the solution. The resources must work adequately, together, and they must be used properly for objectives to be achieved. Beyond the operations of the local team of teams, Ed and Ben go online to buy all sorts of assistive devices (e.g., sleep monitoring, fall detection devices, wrist worn wearables to measure biometrics, smart pill box and usable apps) to help monitor Mary’s path to regaining safe mobility and activities of daily living. And then there are the food deliveries from Ben. It’s quite a logistics and transportation operation. It seems like boxes are arriving at the door every day, sometimes at John and Jennifer’s home nearby. This online shopping spree involves a kind of experimentation with Mary in which her children are playing the time-consuming role of investigators. In this role, they determine the combination of information and services that can help maintain Mary’s ability to remain in the home.

Given the current healthcare provider system, it seems as though the family is constantly wrangling with a wide range of healthcare providers including Mary’s primary care physician and cardiologist who know her well and with more recently added specialists that include a neurologist and neuropsychologist who attended to Mary in an institutional setting before she returned home. Jane and Ed coordinate with weekly caregivers from the Visiting Nurse Association including a registered nurse, occupational therapist, physical therapist, psychologist and home health aide as well as the coumadin nurse and all the other remote specialists (nutritionist, geriatrician, orthopedist, etc.) who must weigh in about Mary’s medications and health status. Beyond medical personnel, they utilize various community services such as the town’s extraordinary senior center. Jane innovates on-the-fly with all sorts of memory aids in the home. She runs the financial operations as well. This includes mind-numbing coordination with various insurance providers and healthcare administrators. Fortunately, both Jane and her husband Jalen, work in law firms. They are undaunted by bureaucracy and answers to questions that often are thinly disguised guesses and run-arounds.

All the family caregivers keep in regular contact via text. Ed collates it all in a running journal that he makes available to the others online in “the cloud.” These collaborative journal entries, which are just texts to most of the family, include observations of Mary’s condition via the internet cameras and video conferences. This enables Mary, somewhat improbably given her persistent cognitive impairment, to live alone after a few months. The family doesn’t have to tag team anymore by staying overnight in the upstairs bedroom. The technology allows for continual assessment of Mary’s physical and cognitive capabilities over the course of a few years. This seems like telehealth, but medical personnel are not directly involved. Experimentation occurs on a daily basis in homes but without the formality of science and outside the reach of evidence-based medicine and the constraints of third-party payers. The effects of this personal initiative on assessment and intervention are largely undocumented outside of the institutional settings that strive for continuity of care across fragmented medical specialties.

Beyond the technology, embedded observation also is important. Ed and Ben develop a more nuanced understanding of Mary’s life during weeklong stays in Mary’s home. As miraculous as Mary’s physical recovery seems to outsiders, it is clear to Ed and Ben that there are rather significant day-to-day variations in her cognitive and motor capabilities. There is natural variation within Mary’s day as there is for any person. Much of this seems linked to diet, sleep and exercise, but there are other variations that are vexing and require a kind of constant reorientation to what Mary is capable of. This is Mary’s daily reality. Now, at 93-years-old, she must adapt almost continually as some functions recover while other functions don’t improve and even show further degradation, the most worrisome being her cognitive status. Ed notes that Mary is engaging in a kind of deliberate practice at adaptation itself. She maintains a daily regimen of routine, for example, that helps to minimize confusion episodes and allows her to remain living independently in her home. He muses that the resulting skill that his family can help Mary acquire turns the common notions of fluid intelligence on its head. It changes their view of aging, now thinking of it as a skill of adaptation and resilience. This changes everything, and the experiment continues.

It is easy for the *ad hoc* team of teams to become overwhelmed by the ever-changing present. As their self-organizational experience and skill in continuity of care improves, they become more aware of the need for future planning, in a sense, for different missions. Mary and her family are again fortunate in that Mary spent the latter part of her nursing career in assisted living complexes that included multiple venues to accommodate various levels of independence and skilled medical care. This background will help them become informed consumers of such services which are expected to evolve rapidly in society. When and how will her requirements for daily and weekly in-home support change? When will it be unwise for Mary to continue to live on her own? What kind of facilities and devices will be needed for her to participate in the activities of daily living as she transitions into more assisted living? How will her life change when and if she requires skilled nursing care more continuously? How can she minimize the loneliness of relative isolation from her extended family and friends when she is not in her own home where she has been surrounded by memories of a life well lived? Will there ever be an end to the need for her continually to relearn and adapt to her changing capabilities and environment? Should there ever be an end to this need to learn and its relationship to a meaningful life? And how does this all relate to end-of-life planning, preparation and awareness?

What happens to the Marys who don’t have this kind of support system in daily life and in planning for the future? For those fortunate ones, much is required of the caregivers. In Mary’s case, caregiving by an *ad hoc* team of teams was sufficiently comprehensive that it filled a common gap in continuity of care by trained compensated healthcare providers. Like most families, Mary’s caregivers had to learn on the job, so to speak, with little or no formative feedback from experts. It is working, but it is precarious. Still, it reveals the art of the possible. It reveals the need for game-changing technologies that, for the most part already exist, some specifically designed for healthcare and others that are not. And what about the lone caregivers whose dauntless endurance [5,6] occurs somewhat in isolation, outside the awareness of most of society and certainly beyond the awareness of healthcare systems and third-party payers?

**Figure 1.**
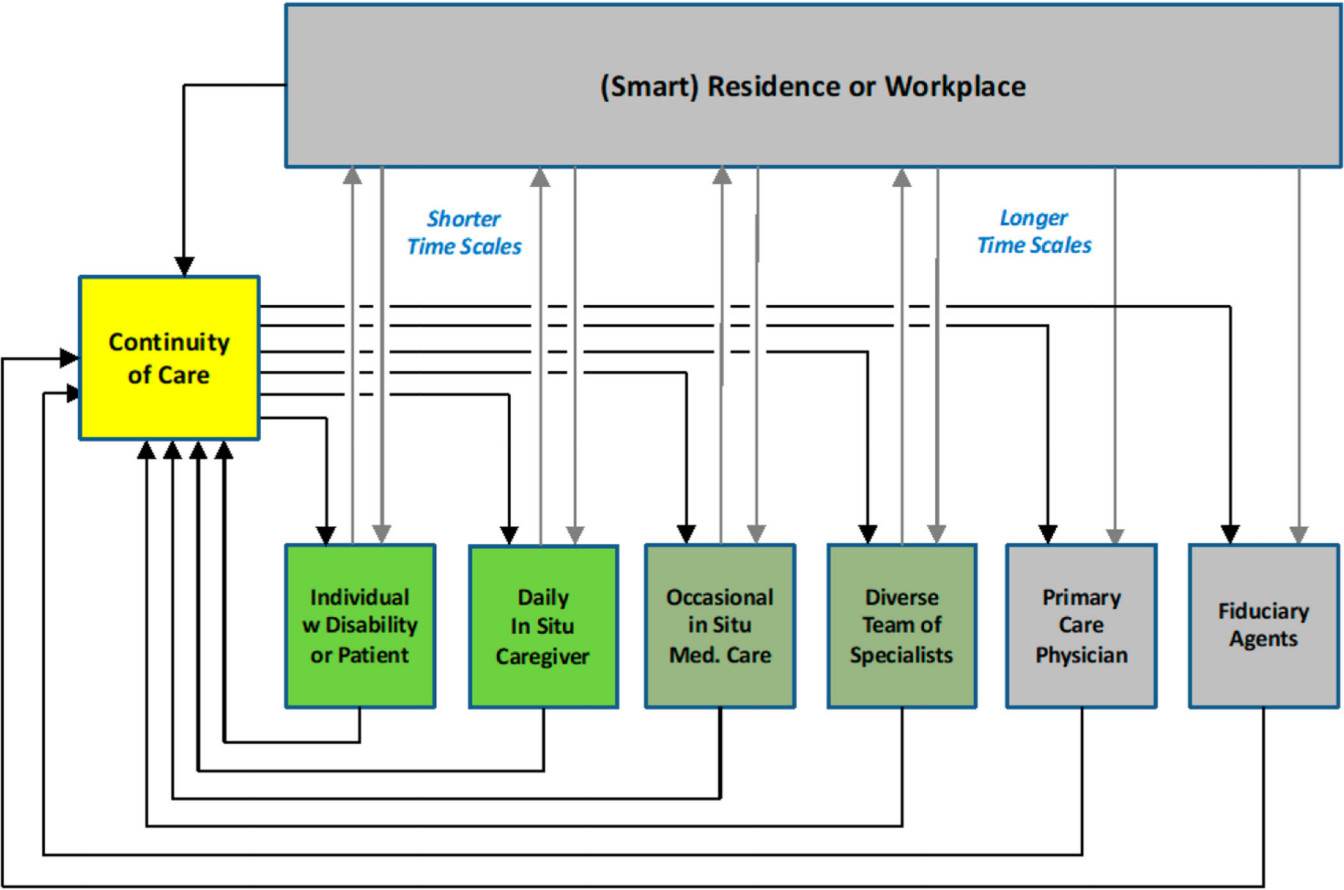
Continuity of care involves coordination among different kinds of stakeholders who typically are engaged intermittently over nested time scales ([3], compare with [4]).

A critical impediment is the lack of a modular systems architecture to ensure that everything works together and that allows for upgrades and continual experimentation with a variety of products. Moreover, such systems architecture both implies and constrains an operational architecture that guides coordination across the team of teams (Figure 1). We believe that the solution for lone caregivers as well as the team of teams from which people like Mary benefit is a smart medical home that can make continuity of care more comprehensively transparent, intelligible and thus systematically improvable. A smart medical home is a tangible construct that broadens the perspective on “study design/data collection” and that can guide development of new paradigms for the “entire continuum of brain aging” [7].

### Translational Research for Health and Wellness

**Figure 2.**
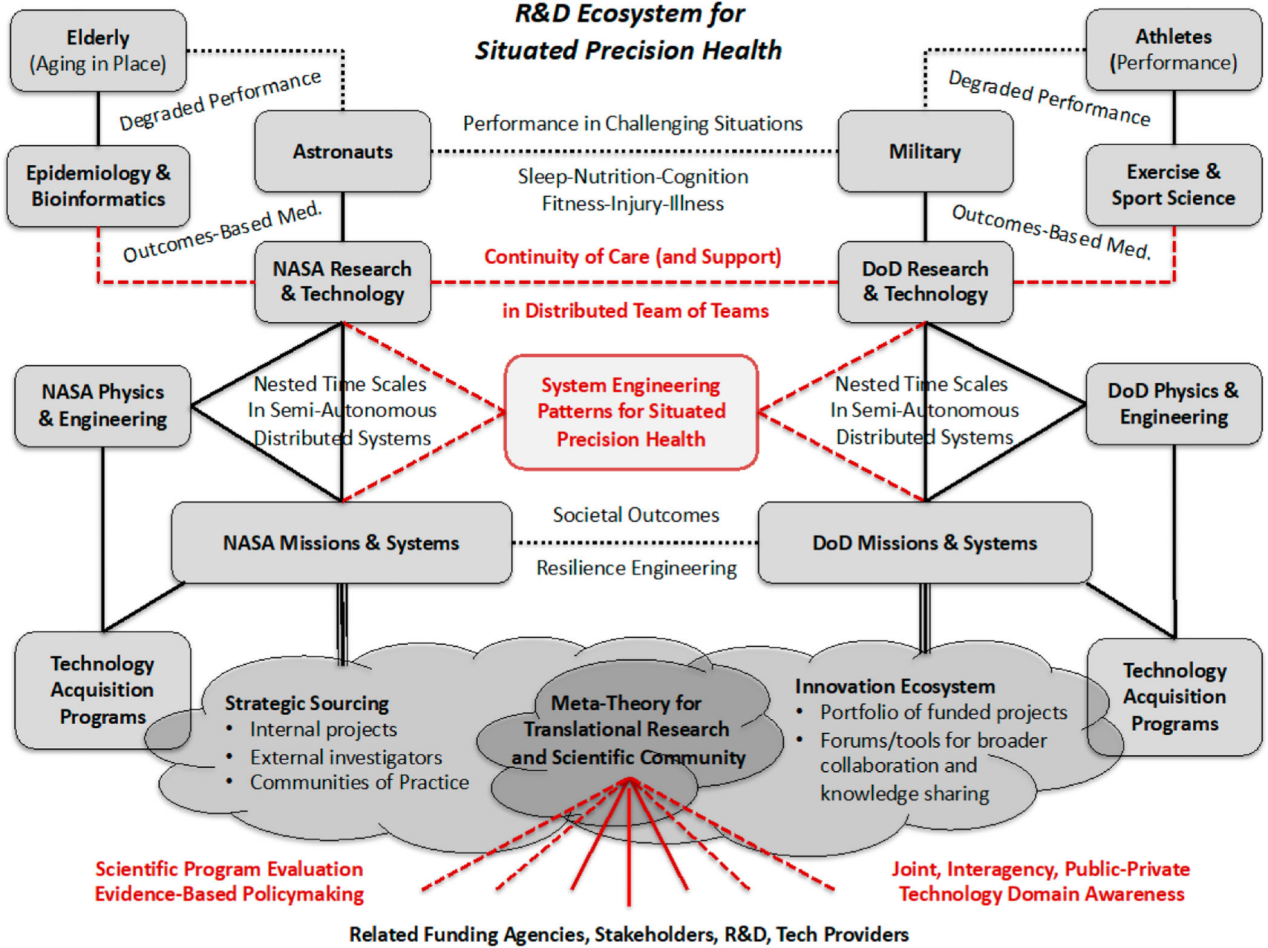
Framework for knowledge transfer among our disparate communities of research and practice. ([9], compare with [10]).

Au called for “discussions tapping into other disease states to better understand their relationship to brain aging… [and] to aggregating data that could help us embrace the full complexity of dementia/AD” [7]. In this article, as in our work with NASA [8], we reveal broader challenges and opportunities for aggregation. One is the aggregation of methodologies from different scientific and technical disciplines that typically aren’t utilized concurrently or in a coordinated way. The other is aggregation of research communities that rarely enjoy a productive dialectic and that may even be unaware of each other. Relevant research in the military and exercise sport sectors are not discussed in this article but our overview of NASA’s approach to research in human health and performance generalizes to work in those communities and benefits from it (Figure 2). The three levels of aggregation (data, methodology, community) are inseparable when research is motivated by outcomes such as efficacious and expeditious continuity of care to provide value that matters most to healthcare consumers.

Kitson and her colleagues have argued with pragmatic clarity that such holistic approaches to innovation in healthcare are, in essence, challenges of knowledge translation among different kinds of stakeholders [10,11]. Their work situates the observation and intervention of individuals in a network of care providers and influencers. They recognize such complexity and implicitly acknowledge that ignoring complexity does not make it go away. Consistent with the challenge posed by Au [7], they call for new approaches to knowledge translation that create the capacity for the various participants in healthcare to “flourish across complex adaptive systems” [11]. Our intent is to draw attention to methods that can be communicated and generalized across disparate research communities: (a) diversity of experimental analogs for holistic translational research; (b) assessment of consequences of medical status for life and work; (c) Multiscale computational modeling, simulation, and analysis; and (d) scientific focus on use, utility and value at the point of care [8].

### Why NASA?

Although the exotic details of NASA’s missions are quite different from the quotidian circumstances of ordinary people affected by the diseases of aging, many of the challenges to people and systems are similar. As in aging, astronauts must continually adapt to their changing capabilities and environments, becoming more skilled through experience at the same time that some cognitive and motor functions degrade. And they face the risk of acute medical conditions with the threat of injury or death. The support system for astronauts must develop the capacity to be both proactive and reactive in assessing the continually changing risk, coping with deviations in health and performance, and treating medical conditions. Some of the people in this support system are medical personal, but many are not, and the most appropriate expertise is not necessarily accessible in the moment.

NASA’s unique experience, over its entire history, is with the systems architecture and the associated operational architecture in a team of teams needed to support the life and work of astronauts; that is, both health and performance. This experience has led to innovations in translational research, implementation science, and their intersection with systems engineering [8]. In a diverse community of peers, NASA addresses priorities and tradeoffs in allocation of resources to various scientific paradigms and continual assessment of the respective return on investment. This multi-stakeholder decision making yields insights about new lines of research that emerge from continual assessment of experience with extant lines of research. Our intent is to show how NASA’s approach to science program management can guide innovation for a smart medical home, most notably for AD and other diseases of aging.

## IMPLICATIONS FOR LIFESTYLE INTERVENTIONS IN THE MEDICAL HOME

### Lifestyle Interventions Occur with or without Science

Au and her colleagues review evidence for the costs to society of chronic illnesses in aging, including one cumulative cost estimate of $47 trillion from 2011 to 2030 [12]. Such total cost estimates are startling not only because of their magnitude, even allowing for substantial uncertainty, but also because they broaden the field of view to include income losses as well as medical expenses of individuals and because they consider costs to a wide range of stakeholders. Medical conditions have cascading consequences for caregivers, institutions that must make accommodations for disabilities, and for the life and work of those on whom the existence of any individual has an impact with or without a disability. On this broad view, an important distinction between medical care and health care, as suggested by Au, is that the latter situates observation and intervention in the meaningful existence of individuals, that is, in their interrelationships with others and their impact on the world around them [13,14]. While this might seem like a lofty set of philosophical considerations, they could not be more practical in the daily lives of patients [15,16]. This broader context focuses directly on what matters to patients. The question for evidence-based medicine becomes whether science is up to the task of addressing the impact of medical conditions, such as those due to the diseases of aging, and experienced in the meaningful lives of individuals involved. We believe that it is, partially based on the work of NASA in which the outcomes of medical conditions for the life and work of astronauts in space are continually monitored and considered in medical care and in the broader support of their health and well-being in extraordinary situations.

Au et al. [12] emphasize the value of prevention and strategies for increasing clarity of knowledge about risk for individuals as well as populations. They also describe the impediments to holistic risk management when there is poor coordination among stakeholders whose interests are not aligned in an obvious way. In our view, the path to a solution starts with a theory of who should be talking with whom about what, as well as how and when to do so, and why it is necessary (Figure 1). The observations of Au et al. about the potential of setting-based approaches, if not the necessity for them, are echoed in NASA’s model-based systems engineering (MBSE) for the design of habitats for astronauts [17,18] (discussed below). NASA explicitly recognizes, of necessity, that settings of work and life are rich sources of people, systems and events that can be observed and influenced through interventions. As suggested in the vignette above, consumers will engage in their own lifestyle interventions, and they experience the effects of them, whether healthcare providers are aware of it or not, and whether providers approve of it or not. In a sense, setting-based approaches seek to harness this external problem solving and intervention that otherwise can be vexing to healthcare providers.

Au et al. highlight evidence about some of the more promising lifestyle interventions such as diet and exercise [19,20]. The effects of such interventions are difficult to disentangle in common paradigms [21]. Diet has been shown to improve cognition in older groups [22], for example. And while the positive effects of exercise on cognition in older adults are well recognized, interventions that combine exercise and diet specifically aimed at Type 2 Diabetes in older groups are equivocal in qualitative data collected over ten years [23]. Such landmark epidemiological studies are exceedingly valuable because of their singular accomplishments in collection of longitudinal data on process and outcomes. At the same time, the quality of data from self-reports and interviews data is highly uncertain, and they are replete with confounds (e.g., unreported and unobserved activities) that may be intractable analytically.

Retrospective qualitative data are impossible to reconcile with quantitative data showing that white matter hyperintensities (medical imagery that suggests latent or manifest demyelination and axonal degeneration) in the brain are reduced in groups with Type 2 Diabetes by lifestyle interventions [24]. Heterogeneity of elderly groups employed in common research paradigms also is a serious problem requiring new, additional paradigms that are more precise, more personalized and more situated [7,12]. Research into interventions must strive to be as multifaceted as the interventions that the elderly and their caregivers explore outside the view of science [25–28]. It is our contention that NASA provides a foundation for development of new paradigms in situated precision medicine because it has the opportunity to observe and influence the life and work of astronauts, holistically, in a smart habitat.

### Introducing Science to the Point of Care

Arguably, NASA flight surgeons and the broader ground support teams have been practicing personalized medicine for decades given the frequency and richness of quantitative and qualitative information collected on astronauts. At the same time, NASA researchers are monitoring the acceleration of capabilities in sensing and computing so that NASA’s personalized medicine can be made increasingly precise and, more importantly, so that it can become more fully situated in the hazardous space environments where astronauts work and live. NASA thus can lead the way in the integration of biological sensing with behavioral sensing and environmental sensing to cross the Rubicon from context-free medical care to context-rich assurance of wellness and performance. There will be vast exploration and innovation in this kind of situated precision healthcare in industry globally [8,12], but NASA offers a unique model for proving grounds to advance the required systems science. This potential has been recognized explicitly in strategic objectives of NASA over the years to benefit life on Earth [29] and in the need for research on human resilience in challenging situations [30,31].

**Figure 3.**
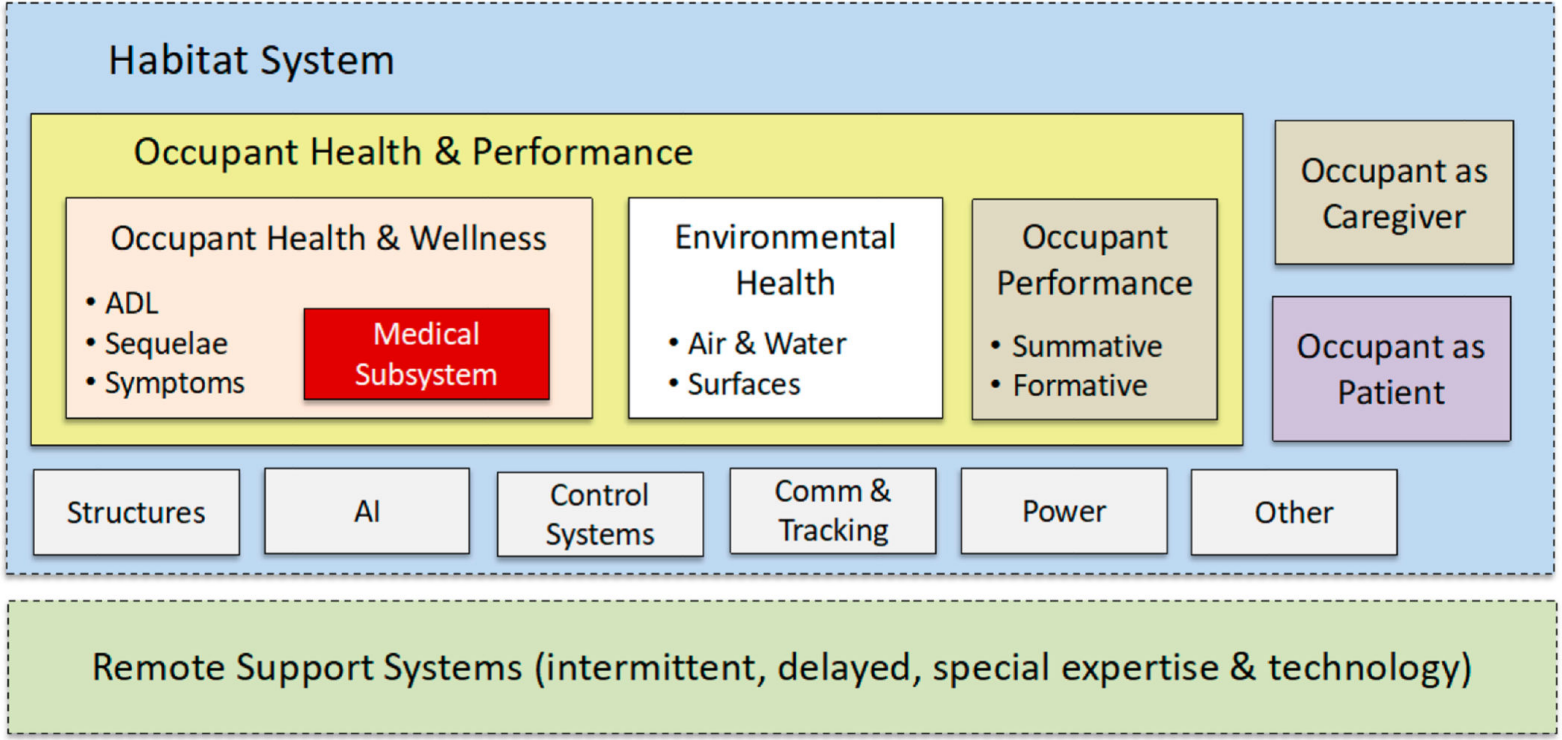
Situating subsystem design and development in nested systems for deep space exploration or a smart medical home (adapted from [34]).

NASA plans and prepares for its grand missions over decades, and it must develop capabilities that won’t become obsolete over those time spans. Industry faces similar challenges even on shorter time scales because of the unprecedented rate of scientific and technological innovation. Resilient architectures for new technology and its use are required to accommodate such change and uncertainty about the future. This is especially the case for capabilities such as precision medicine, autonomy, artificial intelligence, and human-machine teaming that are critical to NASAʼs missions for human exploration in deep space [32,33]. Industry will be able to learn from NASA about resilient architectures as we venture into the fourth industrial revolution [8]. NASA’s model-based systems engineering, for example, is essential in the conceptualization and development of resilient architectures [8,17,18] (Figure 3).

The value of MBSE is that it helps bring science to the point of care. It helps science be sufficiently broad in scope to address the fragmented mosaic of care that individuals receive mostly in the home from healthcare providers who are compensated by insurance companies, healthcare providers who are retained outside the view of such gatekeepers, service providers outside the healthcare industry, friends and family in the role of caregivers, and more-or-less autonomous self-care. As a method of generalization, MBSE also help science be sufficiently flexible to accommodate variation in this mosaic of care within and across situations. While this might seem like a daunting task for science, it should be noted that key decision makers in any mosaic of care already have an implicit theory of who should be talking with whom about what, as well as how and when to do so, and why it is necessary. And they are making decisions that have profound existential impact on quality of life. Anything science can do to demystify this ubiquitous chaos in continuity of care, and to provide intelligible paths for improvement, will be extraordinarily valuable for individuals and for society in general.

### NASA’s Experimental Analogs

**Figure 4.**
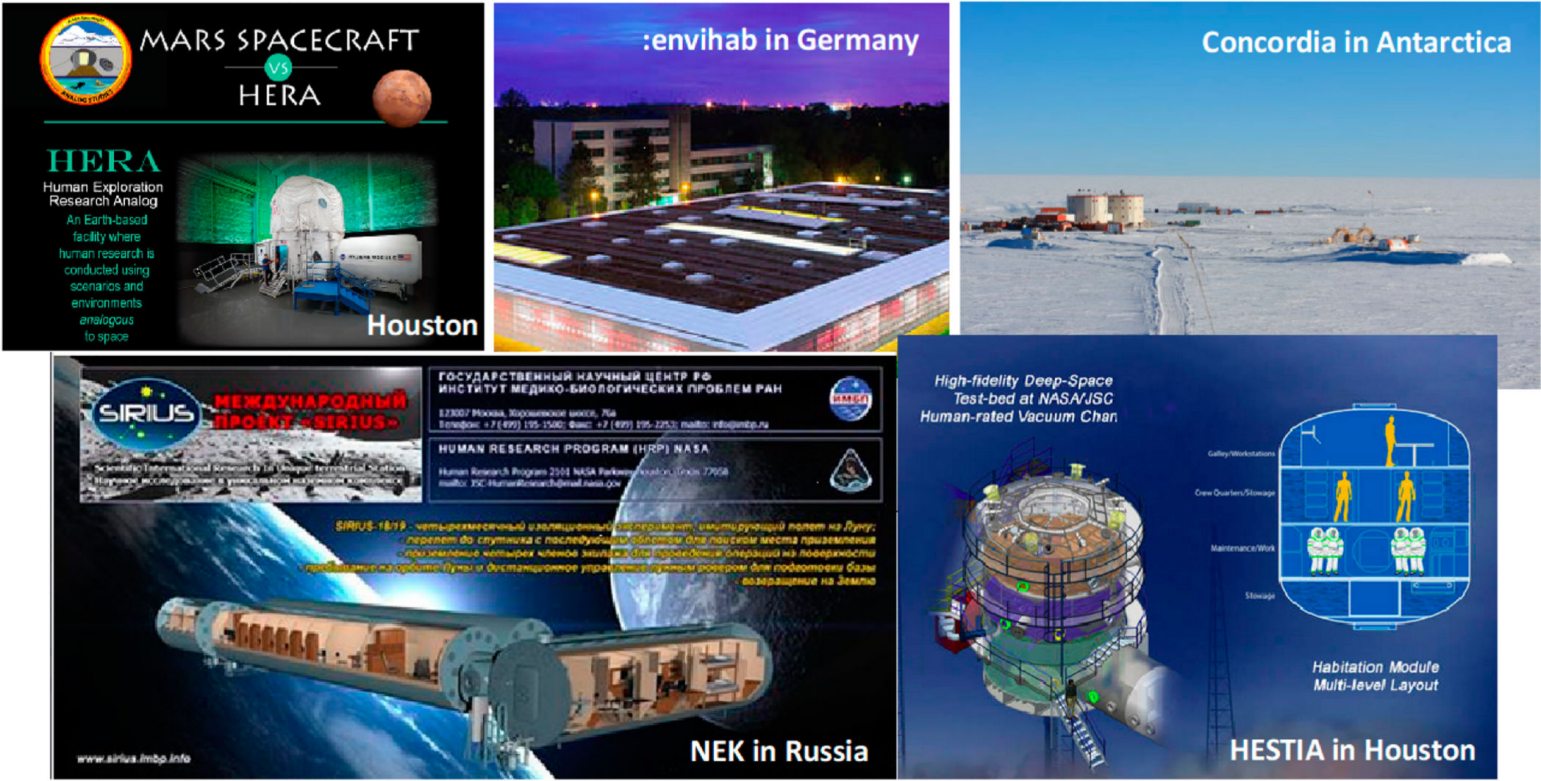
NASA analogs utilized to study the effects of prolonged isolation and confinement (adapted from [35]).

MBSE enables programmatic utilization of a diversity of test beds to be more strategic and coordinated. NASA’s human research utilizes a variety of research settings [35]. The “analogs” enable NASA to study the physiological, psychological and social effects of prolonged shared confinement and the associated continuity of care in remote settings (Figure 4). The NEK facility in Russia provides an infrastructure that enables researchers to assess small teams isolated for up to a year in a habitat that is comparable to plausible deep space habitats. The Concordia facility in Antarctica provides a psychologically realistic environment in that it is inaccessible for months (e.g., “winter over”), as in space, even in an emergency. Through prolonged bedrest (months), the :envihab facility in Germany enables researchers to study conditions that can lead to cardiovascular, respiratory and musculoskeletal problems as well as fluid shifts. The HERA facility at NASA’s Johnson Space Center (JSC) is a highly accessible laboratory for a continual sequence of experiments with crews confined up to 45 days. The HESTIA facility at JSC provides a rapid reconfigurable habitat to assess the impact of design alternatives on occupants.

While NASA’s analogs are as obscure as they are fascinating to the general public, the research on prolonged isolation and confinement of small groups in these facilities has taken on global relevance in the lives of many families at the time of this writing because of the COVID-19 pandemic [36–38]. This wide attention, if not viral media coverage, is helping the general public understand the health effects of prolonged isolation and confinement as well as medical and lifestyle countermeasures for it [39,40]. Such research in NASA also sheds light on challenges of the elderly who are aging in place [41,42]. The COVID-19 pandemic is bringing elder care out of the shadows because of severe impact on this population and because it is providing everyone else direct experience with some of the social and cognitive challenges faced by the elderly including the need for continual adaptation and resilience. NASA’s approach to science at the point of care can accelerate digital and “consumerized” healthcare [8,43–48] that certainly will become even more of a priority because of the pandemic [49].

### Experimental Analogs for Lifestyle Interventions

Intervention can be tested in “real world” conditions in analogs that are sufficiently rich to observe the effects on lifestyle and the effects of lifestyle (lifestyle analogs). The laboratories in the Institute of Applied Life Sciences at the University of Massachusetts, for example, have a level and kind of fidelity commensurate with NASA’s experimental analogs [50,51]. Participants in the research can be observed in situations analogous to a place of residence. That is, they can engage in the activities of daily living in ways that are observable by investigators. Any product or service thus can be *situated* in conditions in which there is the potential for a rich network of cascading consequences of their use. Lifestyle analogs also allow for some degree of autonomy in use of products or services as well as observation of consequences that were not intended or considering in their design. Insights about integration of a variety of products or services can be gained, for example, as research participants bundle them on the fly in the surprising ways that consumers often do. Such opportunities are why situating capabilities integration and development in high-fidelity analogs also is a best practice in the military [52,53].

Lifestyle analogs provide settings and manipulanda that are familiar to a diversity of stakeholders or that can become familiar to them. This setting for collaboration essentially offers boundary objects to facilitate communication and shared experience that can accelerate collective intelligence [53,54]. Situatedness and stakeholder collaboration, together, assure that capabilities integration and development will be informed by empirical evidence about use, utility and value rather than only in terms of elegant synergies among component products or services that may or may not matter to healthcare stakeholders. This is exceedingly important because, even as obvious as the former is as a priority, the latter can be seductive to scientists and engineers who understandably can have deep emotional connection to esoteric ideas and technology that is innovative if not futuristic.

As in NASA’s analogs, lifestyle analogs for situated precision healthcare should be designed with a technical architecture that can be instantiated in a platform to which a variety of sensors can be connected, including those not yet invented or identified (i.e., an extensible architecture). In lifestyle analogs at the University of Massachusetts, for example, special emphasis is given to developing a platform for low-power wireless connectivity to sensors that, among other things, allows for the mobility and non-intrusiveness necessary for the activities of daily living. This paves the way for development, integration and assessment of sensors that can provide measures on multiple scales and of different kinds commensurate with those of interest to NASA [55,56]. Targets include microfluidic biomarkers, wearable sensors for gross performatory movement, subtle motion of biological subsystems, and various physiological signals, as well as environmental parameters (with due diligence on issues of personally identifiable information). The technical architecture for a lifestyle analog must include data collection and archiving that enable the integrative analyses of such heterogeneous data that are essential to identify and interpret meaningful outcomes; that is health and performance in context [34,57].

### Lifestyle Structure and Resilience Analysis

High-fidelity analogs also offer opportunities for data collection about interventions and covariates that influence human health and performance under somewhat naturalistic conditions. We use the term naturalistic to apply to experimental conditions that replicate or simulate the most influential aspects of the physical, chemical, biological and social environment in which a product or service will ultimately be applied. Equally essential to naturalistic conditions is engagement and observation of participants on time scales sufficient to observe common cycles in the activities of daily life or work. This ostensibly simple definition leads us to a generally neglected and relatively untapped source of innovation: temporal structure in the lives of individuals at home or at work. While individuals vary considerably in their adherence to daily routines and to calendars over longer cycles such as weeks or months, there is considerable reliability in cycles of sleeping, eating, exercise, work, rest, and discretionary activity for most individuals. Such variation can be a powerful source of observable perturbations on human physiology and psychology that can be utilized in analyses of interrelationships among measures of different kinds and on multiple scales from molecular to social.

**Figure 5.**
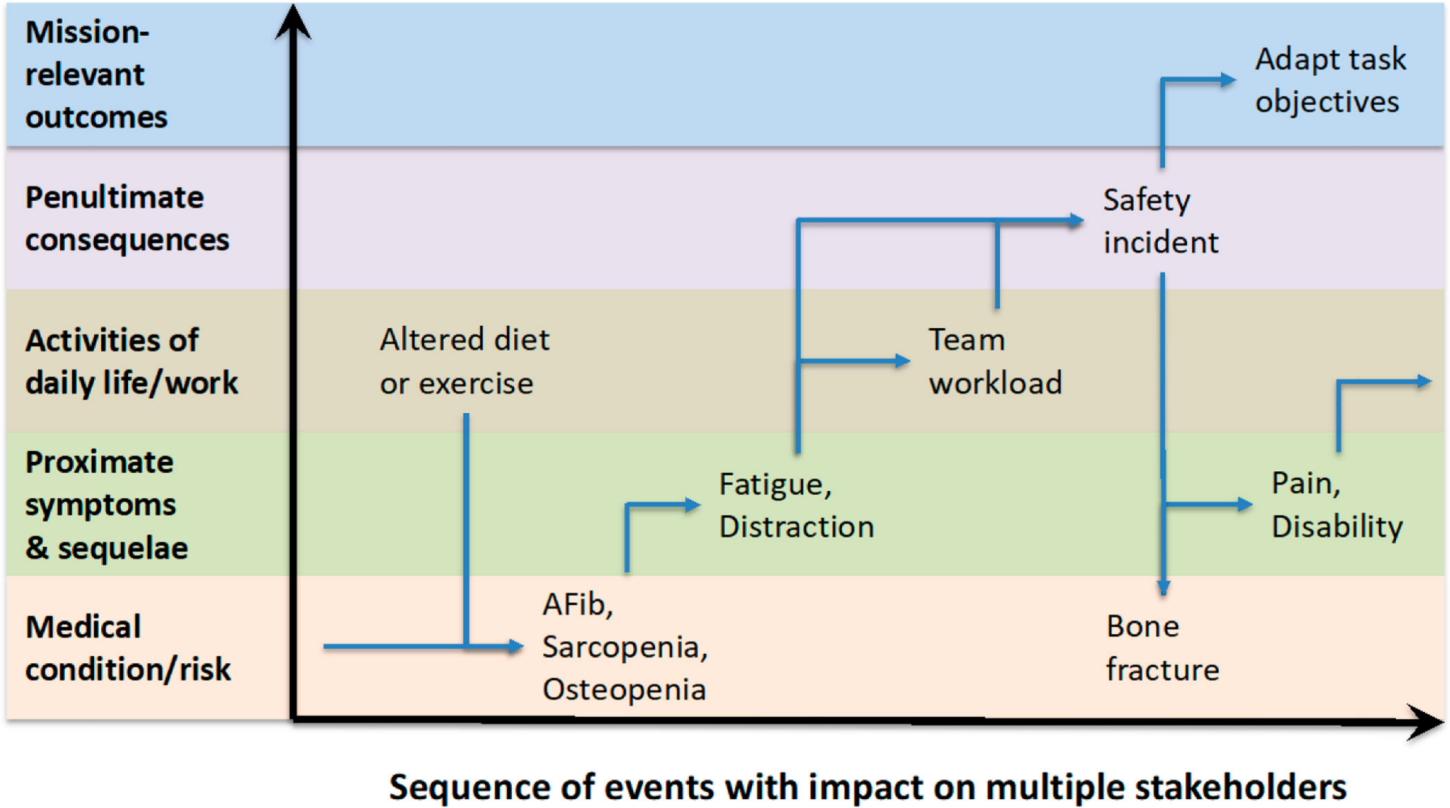
Notional adaptation of NASA’s event-driven Bayesian network modeling and simulation combined with its multiscale modeling [8,58].

Consider, for example, the hypothetical chain of causality and bifurcation in Figure 5 [8,58]. The cycles of physiological status and activity generally lead to natural variation in fatigue, confusion, distraction, clumsiness, weakness, and withdrawal. For example, these variations can have an impact on an individual’s capabilities to observe, orient, decide and act in the activities of daily living. Ultimately, these effects may lead to failures in performance, whether benign or hazardous, and this can have an impact on mortality, morbidity and quality of life in the home [59,60] and safety at work [61,62]. The cascading impact of such perturbations can be generalized to some extent across causes, normal or extraordinary, such as mental and physical overwork, sleep loss, memory loss, pain, anxiety, depression, and medications as well as problems with respiratory, cardiovascular, digestive, musculoskeletal, or sensory systems.

Ultimately the extent that cascading effects can generalize from natural variation to medically noteworthy conditions is an empirical question, but this presents opportunities for development of situated precision healthcare. It allows for a continual failure analysis or, more generally, a continual analysis of resilience [63,64]. In other words, lifestyle analogs provide the opportunity to observe what goes right with individuals in situations that are potentially hazardous or debilitating as well as what goes wrong in such situations (compare with [15,16]). This is not an esoteric consideration peculiar to research. Caregivers and individuals themselves make assessments of efficacy every day, implicitly or explicitly. Medical science must become better informed about such quotidian assessments and the attendant decision-making in place that have an inescapable impact on quality of life.

## IMPLICATIONS FOR MULTISCALE COMPUTATIONAL MODELING

### Meeting the Challenges of Transdisciplinary Collaboration

There is a problem in the inescapable complexity of transdisciplinary science in the era of big data from omics, wearable sensors, and the internet of things that all can and should be collected in lifestyle analogs discussed above. The Life Science Data Archive for NASA’s Human Research Program [65], for example, includes experimental data on physiology and behavior with discrete and continuous variables as well as data on biospecimens, images, mission, personnel, documents, and hardware. The Lifetime Surveillance of Astronaut Health database [65] includes data on (a) population-based clinical care and occupational surveillance, including evaluation of exposure histories and follow-up based on clinical protocols, (b) active surveillance for conditions of interest, (c) Space Medicine operations and countermeasure effectiveness assessment, (d) analyses supporting development of requirements for human-rated vehicles, (e) the Human Research Program gap assessment, and (f) transition to operations assessment. The time scales for these data range from minutes to decades, and the sampling rates vary from fractions of a second to years. The data are structure and unstructured. An effort is underway to define a suite of “standard measures” from which proposed experiments can select to facilitate *post hoc* meta-analyses across investigations and over time [55,56]. Standard measures will evolve as evidence is gained about their use, utility and value.

The qualitative and quantitative complexity of transdisciplinary data that can be collected and analyzed with increasingly sophisticated technology is outstripping the development of commensurate theory and dialectic in the scientific community [66]. The solution is to reduce reliance on the competencies and time of people to identify mutually relevant context, models, underlying processes, emergent properties, and their implications for action across scientific disciplines [67]. The need for solutions to such collaborative overload is being recognized more broadly in business [68,69]. Both science and business have an increasingly urgent interest in developing and integrating capabilities for computational reasoning (e.g., machine learning) that can help overcome collaborative overload in the fourth industrial revolution. Progenitors for situated precision medicine in the smart medical home must participate in this innovation in human-machine collaboration or at least become informed consumers of it [8].

### Cross-Cutting Computational Modeling

NASA’s cross-cutting computational modeling project (CCMP) is engaged in a broader scientific community of multiscale modeling and analysis [70,71] while it is also intimately intertwined with NASA’s model-based systems engineering teams for development of exploration medical capabilities [18]. This multi-faceted multi-layered approach to collaboration recognizes models as falsifiable scientific theories that can develop reciprocally with the accumulation of evidence from interactions with a diversity of stakeholders (see sections below on use, utility and value of research and technology development). The value of such computational modeling is partly that it makes the assessments of various medical conditions and associated risks more commensurable with respect to outcomes. Beyond this source of insight, such models incorporate hypotheses and findings about the downstream consequences of risks, individually and collectively, as well as factors influencing both the likelihoods and consequences of risks [72]. With this objective in mind, NASA utilizes Bayesian network modeling for longitudinal analysis of sequential and coincidental events (i.e., network of events) involving the same group of participants and investigators.

Consider, for example, observation of a sequence of events that starts with a broken exercise device on ISS [70]. Lack of exercise combined with lack of load bearing in weightlessness (as with prolonged bedrest on Earth) leads to loss of bone mass with increased calcium spilling into the blood. Hypercalcemia, in turn, increases the risk of kidney stones. If such a painful and debilitating condition occurs, workload will have to be distributed to other members of the crew. Overwork of another astronaut (e.g., physically demanding activities in a “space suit” outside the vehicle) could lead to a hip fracture because of osteopenia that results from her lack of exercise. If the work that had to be done by astronauts outside the vehicle was necessary either to make a critical repair or for construction that was the purpose of the mission, the potential result is that the mission becomes a partial or complete failure. Bayesian modeling allows planning for such contingencies to be more objective, auditable and improvable. Again, even though the context of spaceflight is exotic, the network of causal connections is not substantially different from the occurrences that must be considered in providing continuity of care for the well elderly who are aging in place and for the increasing populations who will be situated in a smart medical home.

Bayesian network modeling is extensible to causal influences across multiple scales as well as across sequences of events. There will be opportunities to explore relationships across molecular, physiological, biomechanical, behavioral, and interpersonal levels and kinds of measures. We believe it will be more useful to conceptualize these levels in terms of objectives hierarchies (e.g., levels of abstraction) than in terms of the more or less arbitrary boundaries between scientific disciplines [8,73]. Figure 5 depicts a variant of the event sequence described above [70] that utilizes levels of abstraction for a cascade of consequences similar to those described by other NASA investigators over the years [74,75]. The practical relevance of such a means-end hierarchy in a Bayesian analysis of event sequences suggests opportunities for observation of early indicators as well as early interventions and adaptation that can help assure mission success [58]. The implications of this approach for resilience engineering in situated precision healthcare are addressed in the section below on a *lifespan approach to precision brain health.*

### Meeting the Challenges of Big Data in the Smart Medical Home

Implicit in the work on Bayesian network modeling for longitudinal multi-scale analysis is that computational models make assumptions and generate hypotheses [8]. In general, however, machines and computational models are viewed within the scientific community today merely as tools, albeit exceptionally powerful and indispensable ones. At the same time, government and industry are making extraordinary investments in the capabilities of machines to discover causal relationships ahead of theory generated by humans (e.g., [67,76]). Without a vision of a deeper role for computational reasoning in science, progress in its adoption and impact will be impeded in government, academe, and industry. The opportunity cost will be the inability to “facilitate big hypothesis generation and accelerate discovery by correlating data across scientific domains” [67].

**Figure 6.**
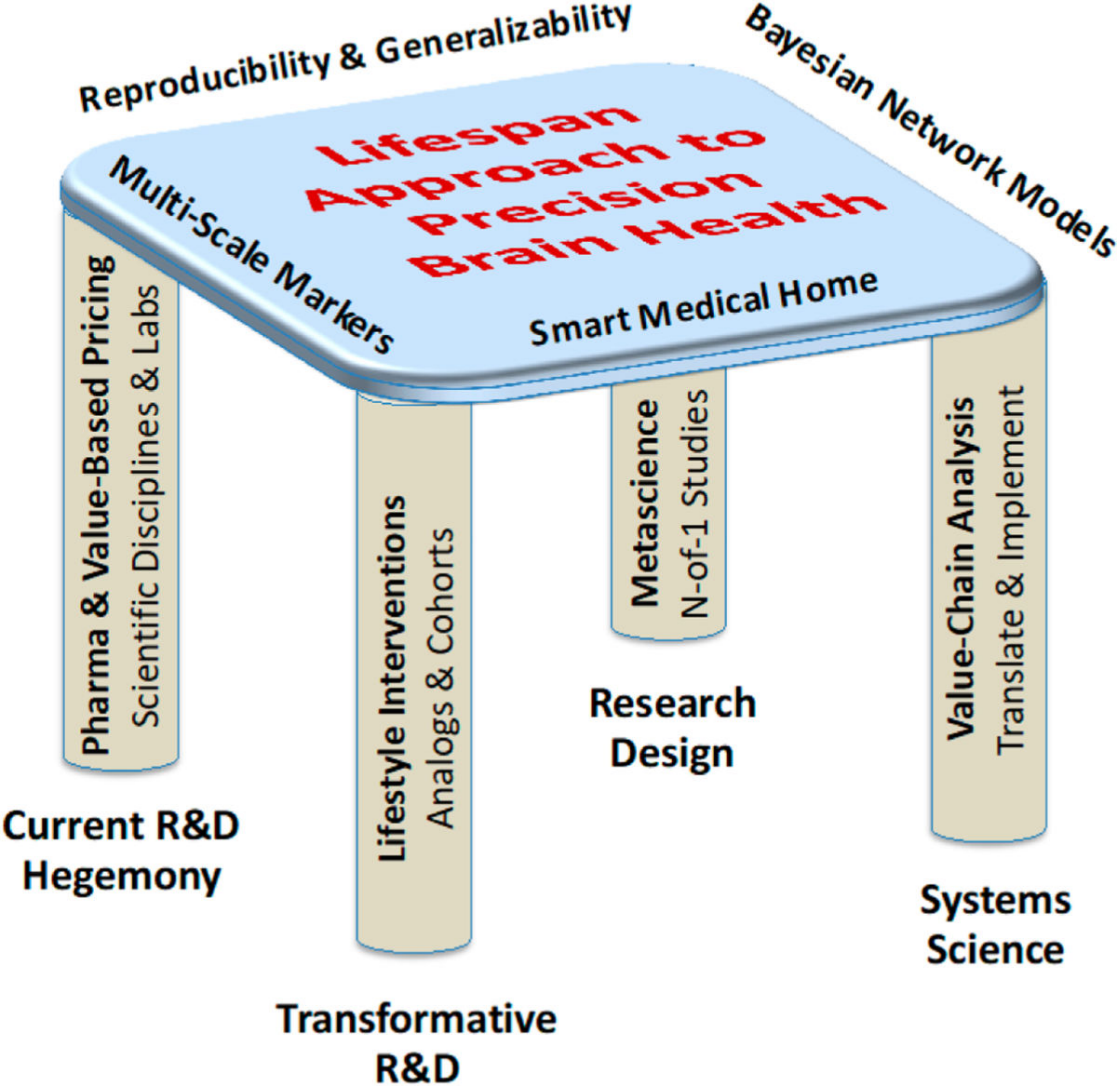
Four pillars of a transformative mission for a lifespan approach to precision brain health (adapted from [82]; compare with [28]).

Lest “participation” of machines in decision making within a scientific community seems exotic, it should be noted that machines already influence what people do in many ways [77] through the information that they make more or less accessible to people and to some extent on the basis of what machines can come to know about the interests, activities and social network of an individual [78]. Closer to our interest in continuity of care for an individual patient, the science of medical decision-making boards is maturing, such as tumor boards and multidisciplinary cancer meetings [79] as applied to genomics [80] and, more specifically to the intersection of genomics and bioinformatics in precision medicine [81]. An extension of human-machine decision-making boards to the medical home is eminently practical and timely given the current state of practice in precision medicine and especially in well-funded specialties such as oncology. This represents a commitment to improvement in evidence-based medicine that can improve quality of life by embedding science in the life of an individual. In the final sections, we take this philosophy to its logical conclusion by emphasizing the importance if not the necessity for a lifespan approach to precision brain health [7] (Figure 6).

## IMPLICATIONS FOR LIFESPAN APPROACH TO PRECISION BRAIN HEALTH

We believe that precision medicine boards are a model on which to build even if it requires a fundamentally different instantiation of them for general use. We believe that utilization of computational reasoning in precision medicine boards, for example, will be required for scalability and affordability. We also believe that *bringing science to the point of care* is required. On this view, the daily longitudinal care of any patient can become a kind of N-of-1 study in which generalizability and predictability, while not limited to an individual, is focused on the individual and thus on the richness of context that can be addressed only at the level of an individual.

### Continuum of Brain Aging

Au et al. [12] conclude that studies are needed to reveal the opportunities for early detection and intervention with respect to modifiable risk factors. They juxtapose this recommendation with the observation that the potential for such work is vastly increased by “setting-based approaches” aimed at *health and wellness* to complement research on *disease* per se. In the call for papers for this special issue, Au specifically emphasizes the importance of “studying dementia/AD outside the boundaries of what is presumed to be known about the disease” [7]. This implies the need to look for noteworthy “change well within the range of normal” and even a different way of thinking about what it means to be “asymptomatic” or “presymptomatic” as well as the points at which interventions should be considered and evaluated [12]. We believe NASA’s broad portfolio of research and technology development provides a framework and key methods for early detection through continual risk assessment and mitigation.

Our approach requires that progressive refinement in the understanding and mitigation of risk take place in situ, either in the settings where patients live and work or in analogs with well-defined similarities and differences with respect to these settings. Scientific investigations should be brought to situations where medical problems and the outcomes of associated interventions matter to patients. Moreover, we suggest a resilience engineering orientation in which caregivers and providers can observe what goes right with individuals in situations that are potentially hazardous or debilitating as well as what goes wrong in such situations. We believe this is an approach that achieves the requisite focus on health vice disease and healthcare vice medical care [7,12].

Our assumption is that the cascading multiscale effects of AD-related impairment in asymptomatic situations can be generalized to other situations, most notably, those in which symptoms of problematic medical conditions may be observed. Of course, this must be viewed as a working hypothesis. Over time and experience with an individual, the extent and limits of generalizability can be determined. That is, longitudinal analysis of individuals will provide evidence about the predictive reliability of early indications of multiscale cascade failures. To be sure, methodological approaches for such N-of-1 studies can and should be refined across individuals through small clinical trials or larger cohort studies, but the focus here is on longitudinal analysis of the individual. In a sense, we are suggesting that science must take the perspective of a primary caregiver or a primary care physician at the same time that the perspective of the latter becomes more scientific at the point of care. Setting-based approaches are much more feasible when focused on an individual, and they suggest an intersection of personalization and precision in healthcare. Unlike personalization that focuses on traits and precision based on increasingly reductionistic science, but in addition to it, we are arguing for a situated precision healthcare in which personal patterns and proclivities are an emergent property of the mind, body and environment [8,82]. We believe situated precision healthcare is a systems approach that, as such, has the best chance to be predictive, preventive, personalized and participatory [83,84] (see e.g., Figure 6).

The continuum of brain aging—viewed as resilience to cascading effects of impairments—can but does not necessarily illuminate the original cause of a medical condition. In fact, it does not assume a single cause at all, although it allows for the possibility of such liminal cases. It recognizes that neurodegeneration and other changes in brain structure and function generally are diffuse and thus affect many aspects of human physiology and behavior. It represents a radical re-centering of healthcare without rejecting more traditional approaches to medicine. It is not a reactive endeavor with an emphasis on remediation or cures often driven by isolated causes and constrained by their relevance and validity. Instead, it gives priority and precedence to proactive vigilance about the healthiness and fragility of a system (i.e., a person in particular situations) that emphasizes awareness of and improvements in resilience. The emphasis on resilience erases the distinction between prevention and rehabilitation in that continual failure analysis informs both. Continual experimentation (nondestructive evaluation) with prevention of cascade failures informs the development and use of rehabilitative interventions, and the capacity of the latter to reduce the likelihood and consequence of cascade failures is, in essence, preventive. The continuum of brain health is neither about traits nor states. It is about personal efficacy and multiscale functional interrelationships that are persistent but not immutable. It starts with outcomes that matter to patients and to those with a stake in sustainability of the patient’s lifestyle or changes in it.

The cascade effects of impairments, whether due to neurodegeneration or not, can be a source of innovation and development of new interventions. It also can be a source of combinatorial innovation that bundles extant interventions to be synergistic or at least noninterfering [8]. It recognizes that patients and caregivers are intervening in their health and well-being, whether intentionally or not, and whether or not in view of institutional healthcare providers. These externalities must be observed and harnessed in a coherent approach to systems healthcare that attempts to nudge the patient toward choices that are healthier or for which there is greater confidence about health outcomes. In any case, surveillance about the impact of as many interventions as possible, intended or otherwise, will be essential in the development of situated precision healthcare in the medical home (see sections below on *use, utility and value of research and technology development*). The accelerating innovation in technology that can be utilized in a smart medical home can facilitate development of such assessment [12]. Continual holistic assessment of challenges and responses will be extraordinarily beneficial to patients with AD and others suffering from diseases of aging who otherwise exist in a chaotic confluence of benevolence, neglect, and ignorance.

### Use of Results from Research and Technology Development

NASA has systems and procedures to assess the use of deliverables from research and technology development. There are human-systems standards, for example, that address crew health [85] as well as human-factors, habitability and environmental health [86]. NASA research informs these standards that are intended for use in decisions about design and development of space systems. NASA also has requirements for the kinds of stakeholders that should be involved in communication about their various standards, collaborative interpretation of them and when they should do so, such as in the context of design review milestones as well as in continual updates of requirements documentation [87,88]. The high-priority processes—in which numerous personnel are involved from across the NASA enterprise—do not include the kind of hypothetico-deductive activity that is characteristic of a scientific community, however, nor is that the intent. This is an opportunity for any research and technology development community that seeks to build on NASA’s foundation by adding scientific inquiry into the methods and outcomes of science itself.

**Figure 7.**
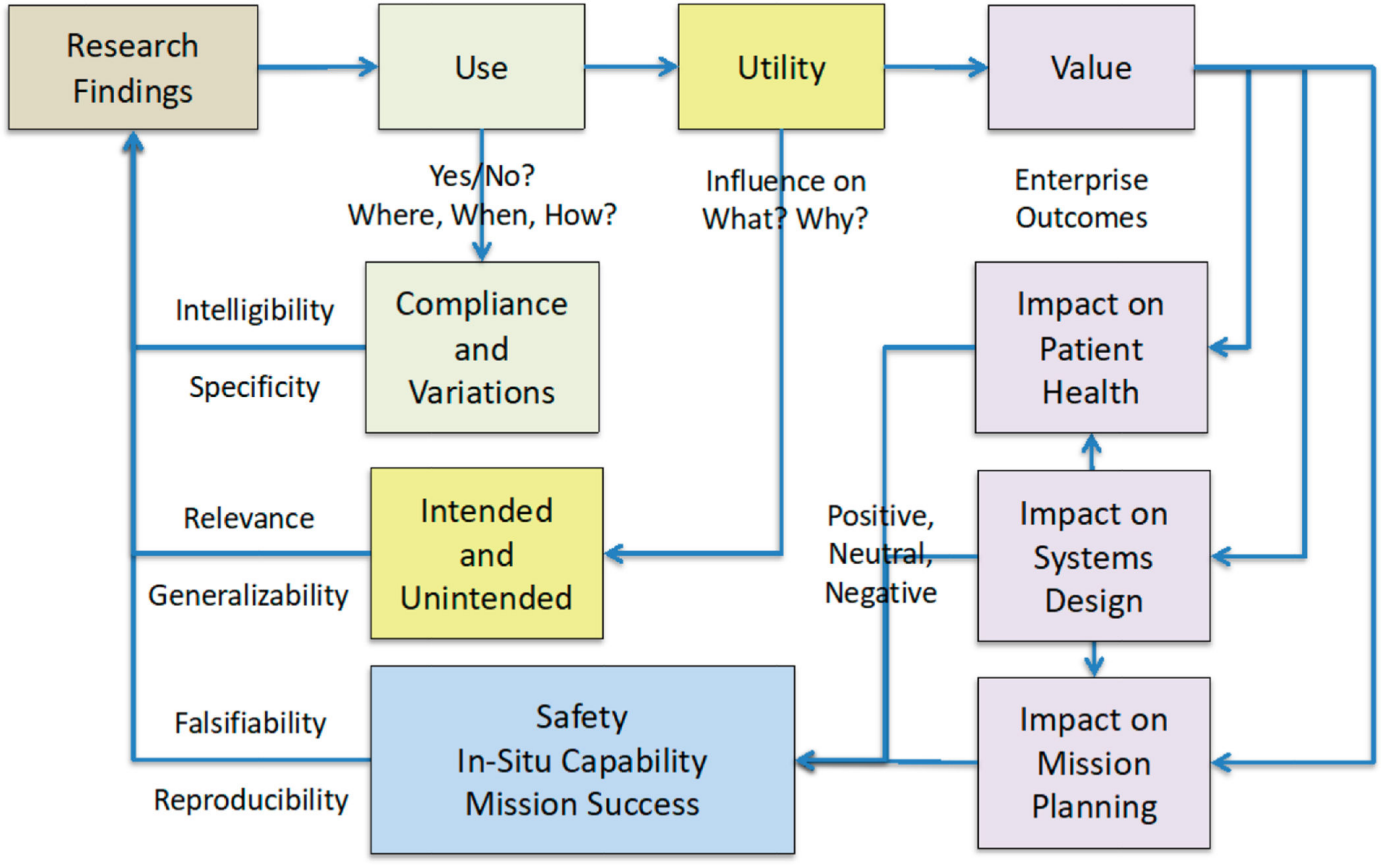
Evaluation of attempts to influence naturally occurring events.

Figure 7 is a high-level logic diagram for inquiry into efficacy (impact), efficiency (affordability), and expeditiousness (timeliness) of research with respect to the needs of multiple stakeholders such as in outcome-based medicine. Consider, for example, the prescription for exercise to mitigate osteopenia and sarcopenia among other physiological and psychological conditions [32,33]. If facilities and equipment allow for exercise in the habitat (home), the first question to ask is whether healthcare consumers actually engage in the prescribed exercise. If they don’t, then the explicit theory of *use* is falsified or otherwise demonstrated to be incomplete. One must identify the assumptions that are vitiated and those that can be retained in a revised theory. Such lessons learned rarely are binary. Users may engage in more or different kinds of exercise as a result of the prescription but deviate from the prescription. The lack of compliance may simply be due to a lack of intelligibility in the way the prescription is communicated through the value chain and ultimately to the consumer, or it may be due to a lack of diligence or skill. In either case, the prescription should be revised, and further research may be needed.

### Utility of Results from Research and Technology Development

Lack of compliance also may reflect extra-procedural activity of the consumer that reveals equivalence classes of activity previously not considered in the research that led to the prescription. Consumers may actually come up with a variant of the prescription that is superior because of idiosyncratic or common conditions that had not been considered in the research. For example, consumers may utilize different equipment or ranges of motion that require less volume. The time savings or accommodation of other activities may be important to consumers but not something that exercise researchers would have considered to be in their span of expertise, responsibility or control. Such information about multifaceted *utility* nevertheless would be instructive for new directions of research that would have clear implications for designers of equipment and the habitat. Such inquiry may even require organizational changes or collaboration among previously independent units to engage in a new kind of transdisciplinary research that responds to the cross-cutting needs of consumers. In general, there is no reason to believe that consumer needs map in any simple way to technical disciplines or organizational units that developed under a different set of selective pressures often dominated by compartmentalized supply-side considerations. This is only a tiny sample of the kinds of actionable information that can be gained by observing use of research products in situ.

As use of research products is verified, there almost always is further clarification of the consequences of use. Consider again, for example, an exercise prescription. Is the effect on bone loss and muscle strength similar to what was observed in a different environment (e.g., laboratory) or otherwise different from what was predicted? In any case, some variations in the effects may be influenced by external factors from the perspective of research that delivered the exercise prescription. Perhaps consumers didn’t comply with a synergistic prescription for bisphosphonates. The reasons for this may lead to modifications of the exercise prescription if compliance cannot be guaranteed in the future. Collateral effects of exercise on sleep and nutrition also may become more refined in the application environment (home). While not surprising, evidence about such complementary activity can lead to changes in the exercise prescription. It may necessitate additional research that could not have been anticipated, and the new line of research may be a higher priority that what had been planned.

### Value of Results from Research and Technology Development

It also is possible that the utility of a research product, such as an exercise prescription, is not entirely positive and in ways that could have been anticipated (i.e., outcomes or *value*). Perhaps the exercise necessitates changes in activities of daily living that involve hygiene and clothing because of perspiration and comfort. Exercise in the application environment may give rise to altered patterns of breathing and cocontraction that compensate for instability when more common skills of balance are ineffective. Old skills may be degraded due to medical conditions (e.g., osteopenia, sarcopenia, pain), or they may be inappropriate because of different mechanical constraints in the application environment (e.g., reduced gravity, potential problems of vibration, range of motion). Modification in the exercise prescription would be needed if these differences were to have an undesirable impact on blood pressure, cardiac rhythm, and fluid shifts. This probably would require different kinds of transdisciplinary research and changes in the priority of various lines of research in the portfolio.

Constraints on exercise also may be imposed to accommodate cohabitation because of noise, respiration, perspiration or utilization of common space. This could necessitate modifications to the exercise modality, the exercise devices and the habitat in which the exercise is situated. Alternative designs and associated lines of research always should be considered in a portfolio. Consider the need to adjust to a smaller habitat because of new missions in deep space for which the luxury of spaciousness on the International Space Station is not possible. In deep space, NASA probably will not be able to utilize the exercise devices and prescriptions it developed for station because of more draconian constraints on mass, volume, and perhaps power. A lifecycle upgrade plan for research on exercise can include inquiry that can gather evidence from the unanticipated utility of larger devices that, at least to some extent, can be generalized to smaller devices and thus accelerate their development. These considerations are not unique to NASA. An analogous need for alternatives is salient in the case of a medical home because it is common for patients to move from independent living to generally smaller spaces in facilities with increasingly more skilled care givers, more sophisticated technology, and a broader range of each.

### Toward a New Paradigm

As the examples above reveal, there is a logical progression from inquiry about use, through utility, to the ultimate value of research products. The holistic impact on patient health obviously is the most important criterion of value. The patient or consumer is the primary and most important stakeholder. There are other stakeholders, however, whose contributions are essential to the patient’s health and well-being [89,90]. The engineers who design and develop the habitat as well as the technology that makes it smart also are critical stakeholders. They must think holistically, in terms of a systems architecture, if they are to minimize undesirable unintended consequences and to maximize synergy of capabilities that can be utilized by patients, caregivers and healthcare providers. This cannot be done without a holistic systems science that is commensurate with the system engineering through which its research products will be translated and transitioned to operation, that is, through which it delivers value. Inquiry into use, utility and value of research is necessary for this linkage. Finally, research must deliver value to healthcare provider organizations and the sociotechnical ecosystem in which they can survive if not thrive. In their ecosystem of revenue and cost constraints, providers must have a mission orientation for health and well-being supported by resilient systems [8,48].

There are clear needs to accelerate the translation of science to practice [91,92] and to make such expeditious science more relevant to individuals in specific situations [93–96]. There is an opportunity to address these needs through longitudinal studies of individuals who are aging in place, especially those who are at risk for AD, given the potential of a smart medical home and the continuity of care that it affords [8] (Figures 6 and 7). In developing a new kind of science for this opportunity, there are broad shoulders to stand on, such as the Framingham Heart Study of the National Heart, Lung, and Blood Institute [97,98]. At the same time, the approach recognized by Au et al. [12] and elaborated in this article has the potential to transform this kind of epidemiology and blur its differentiation from experimental science. NASA has been on the forefront of this difficult but important juxtaposition for some time [8,32,34,65,70,99]. NASA doesn’t have all the answers and, given its small budget relative to the healthcare industry’s investments in R&D [8], the agency’s human research can benefit from peers with whom they can explore the art of the possible through collaboration in a broader crosssector community of interest [100,101]. There is an opportunity for NASA and the healthcare industry to make scientific progress faster together than they can alone.

## CONCLUSIONS

This special issue is ambitious in that it calls for strategic transformation in research on AD and, by implication, other diseases of aging. In our response to this challenge, we give special attention to research translation and knowledge transfer from NASA to the healthcare industry. We described how NASA’s approach to research can guide innovation for a smart medical home, most notably for AD and other diseases of aging. Consistent with the intent of this special issue, we addressed how NASA’s approach to science program management provides a foundation for aggregating fragmented research communities, lifestyle interventions in the medical home, and a lifespan approach to precision brain health. We also addressed how NASA’s approach can guide the utilization of multiscale computational modeling as well as continual improvement through a rigorous focus on use, utility and value of research findings. We provided novel motivations and transformative paths to a diversity of specific lines of research, across communities, that would be difficult to discover in common methods of networking within research communities and even through sophisticated bibliographic methods. We thus revealed how knowledge transfer between the public and private sectors can stimulate development of broader scientific communities and achieve a more coherent strategic approach to integration and development of a diversity of capabilities including but not limited to technology.

## References

[1] Montero‐Odasso M, Verghese J, Beauchet O, Hausdorff JM. Gait and cognition: a complementary approach to understanding brain function and the risk of falling. J Am Geriatr Soc. 2012;60(11):2127–36. 23110433 10.1111/j.1532-5415.2012.04209.x PMC3498517

[2] Roitto H, Kautiainen H, Öhman H, Savikko N, Strandberg TE, Raivio M, Relationship of neuropsychiatric symptoms with falls in Alzheimer’s disease– does exercise modify the risk? J Am Geriatr Soc. 2018;66(12):2377–81. 30320427 10.1111/jgs.15614

[3] Riccio G, Shelhamer M, Menon J. Synergy between developers and stakeholders for autonomous medical decision making. In: Proceedings of 2020 NASA Human Research Program Investigatorsʼ Workshop; 2020 Jan 2730; Galveston, US.

[4] Riccio G, Vicente K. Coping with change and novelty in energy facility operations: Recommendations for knowledge-based, extra-procedural problem solving. In: Electrical Power Research Institute Report 1004666. Palo Alto (CA, US): EPRI; 2001.

[5] Carter R. Helping yourself help others: A book for caregivers. New York (US): Three Rivers Press; 1994.

[6] Peterson B. Voices of Alzheimer’s: Courage, humor, hope and love in the face of dementia. Cambridge (MA, US): Da Capo Press; 2004.

[7] Au R. Heterogeneity in Alzheimer’s Disease and Related Dementias. Adv Geriatr Med Res. 2019;1:e190010. 10.20900/agmr20190010 31497779 PMC6731025

[8] Riccio G. Toward Situated Precision Health in the Smart Medical Home: Insight and Innovation from NASA’s Metascience. In Translational Research Institute for Space Medicine. Washington D.C. (US): NASA; 2020. NASA Cooperative Agreement NNX16AO69A.

[9] Riccio G, Eslami M, Au R. Pasteur’s quadrant for cross-domain scientific progress in situated precision health. 2017. Unpublished work.

[10] Kitson A, Powell K, Hoon E, Newbury J, Wilson A, Beilby J. Knowledge translation within a population health study: how do you do it? Implement Sci. 2013;8(1):54. 23694753 10.1186/1748-5908-8-54 PMC3674953

[11] Kitson A, Brook A, Harvey G, Jordan Z, Marshall R, O’Shea R, Using complexity and network concepts to inform healthcare knowledge translation. Int J Health Policy Manag. 2018;7(3):231. 29524952 10.15171/ijhpm.2017.79 PMC5890068

[12] Au R, Ritchie M, Hardy S, Ang TFA, Lin H. Aging well: Using precision to drive down costs and increase heal. Adv Geriatr Med Res. 2019;1:e190003. 10.20900/agmr20190003 31342014 PMC6656386

[13] Maslow A. Toward a psychology of being. New York (US): Van Nostrand; 1968.

[14] Peterson C, Seligman EP. Character, strength, and virtues. Oxford (UK): Oxford University Press; 2004.

[15] Dahlberg K, Todres L, Galvin K. Lifeworld-led healthcare is more than patientled care: An existential view of well-being. Med Health Care Philos. 2009;12(3):265–71. 19101822 10.1007/s11019-008-9174-7

[16] Sundström M, Blomqvist K, Edberg AK, Rämgård M. The context of care matters: Older people’s existential loneliness from the perspective of healthcare professionals—A multiple case study. Int J Older People Nurs. 2019;e12234. 10.1111/opn.12234 31025806

[17] Mindock J, Reilly J, Rubin D, Urbina M, Hailey M, Cerro JA, Systems Engineering for Space Exploration Medical Capabilities. Presented at: AIAA SPACE and Astronautics Forum and Exposition; 2017 Sep 12–17; Orlando, US. Reston (US): American Institute of Aeronautics and Astronautics; 2017. p. 5236.

[18] Hanson A, Mindock J, Okon S, Hailey M, McGuire K, Bardina J, A ModelBased Systems Engineering Approach to Exploration Medical System Development. In: Proceedings of 2019 IEEE Aerospace Conference; 2019 Mar 2–9; Big Sky US. New York (US): IEEE; 2019. p. 1–19.

[19] Ngandu T, Lehtisalo J, Solomon A, Levälahti E, Ahtiluoto S, Antikainen R, A 2 year multidomain intervention of diet, exercise, cognitive training, and vascular risk monitoring versus control to prevent cognitive decline in at-risk elderly people (FINGER): a randomised controlled trial. Lancet. 2015;385(9984):2255–63. 25771249 10.1016/S0140-6736(15)60461-5

[20] Williamson JD, Pajewski NM, Auchus AP, Bryan RN, Chelune G, Cheung AK, Effect of intensive vs standard blood pressure control on probable dementia: a randomized clinical trial. JAMA. 2019;321(6):553–561. 30688979 10.1001/jama.2018.21442 PMC6439590

[21] Zucchella C, Sinforiani E, Tamburin S, Federico A, Mantovani E, Bernini S, The multidisciplinary approach to Alzheimerʼs Disease and Dementia. A narrative review of non-pharmacological treatment. Front Neurol. 2018;9:1058. 30619031 10.3389/fneur.2018.01058 PMC6300511

[22] Lehtisalo J, Levälahti E, Lindström J, Hänninen T, Paajanen T, Peltonen M, Dietary changes and cognition over 2 years within a multidomain intervention trial—The Finnish Geriatric Intervention Study to Prevent Cognitive Impairment and Disability (FINGER). Alzheimers Dement. 2019;15(3):410–7. 30527596 10.1016/j.jalz.2018.10.001

[23] Hayden KM, Baker LD, Bray G, Carvajal R, Demos-McDermott K, Hergenroeder AL, Long-term impact of intensive lifestyle intervention on cognitive function assessed with the National Institutes of Health Toolbox: The Look AHEAD study. Alzheimers Dement. 2018;10:41–8. 10.1016/j.dadm.2017.09.002 PMC5675717 29159267

[24] Espeland MA, Erickson K, Neiberg RH, Jakicic JM, Wadden TA, Wing RR,. Brain and white matter hyperintensity volumes after 10 years of random assignment to lifestyle intervention. Diabetes Care. 2016;39(5):764–71. 27208378 10.2337/dc15-2230 PMC4839171

[25] Brewster L, Tarrant C, Willars J, Armstrong N. Measurement of harms in community care: a qualitative study of use of the NHS safety thermometer. BMJ Qual Saf. 2018;27(8):625–32. 10.1136/bmjqs-2017-006970 PMC6204929 29197824

[26] Teri L, Logsdon RG, McCurry SM, Pike KC, McGough EL. Translating an evidence-based multicomponent intervention for older adults with dementia and caregivers. Gerontologist. 2020;60(3):548–57. doi: 10.1093/geront/gny122 PMC7117621 30304477

[27] Fertalova T, Ondriova I. Non-pharmacological treatment of Alzheimer’s. In Alzheimerʼs Dementia and Korsakoffʼs Disease-Linking Memory Pathology to the Autobiographical Self. IntechOpen. 2019. doi: 10.5772/intechopen.84893

[28] Atri A. Current and future treatments in Alzheimerʼs Disease. Semin Neurol. 2019;39(02):227–40. 30925615 10.1055/s-0039-1678581

[29] National Aeronautics and Space Administration. NASA Strategic Plan 2018. Washington DC (US): NASA; 2018.

[30] Shelhamer M. A call for research to assess and promote functional resilience in astronaut crews. J Appl Physiol. 2016;120(4):471–2. 26472875 10.1152/japplphysiol.00717.2015

[31] Shelhamer M Enabling and enhancing human health and performance for Mars colonies: Smart spacecraft and smart habitats. In: The Human Factor in a Mission to Mars. Cham (Switzerland): Springer; 2019. p. 59–67.

[32] Human Research Program. Human Research Program: Program plan. HRP-47051, Revision D. Available from: https://www.nasa.gov/pdf/503445main_HRP47051_ProgramPlan_508.pdf/. Accessed 2020 Jun 9.

[33] National Aeronautics and Space Administration. The human research roadmap. Available from: https://humanresearchroadmap.nasa.gov/architecture/. Accessed 2020 Jun 9.

[34] Hailey M, Bardina J, McGuire K, Urbina M, Hanson A, Cerro J, Exploration Medical Capability Medical System Recommendations for Gateway. In Proceedings of 2019 NASA Human Research Program Investigatorsʼ Workshop; 2019 Jan 22–25; Galveston, USA.

[35] National Aeronautics and Space Administration. Analog missions. Available from: https://www.nasa.gov/analogs. Accessed 2019 Nov 24.

[36] Davenport C. Even astronauts get ornery: Coronavirus advice from those who have endured social distancing in the extreme. Available from: https://www.washingtonpost.com/technology/2020/04/16/astronautsquarantine-advice-coronavirus/. Accessed 2020 Jun 9.

[37] Cowing K. The Parallels between Space Missions and COVID-19 Isolation. Available from: http://spaceref.com/coronavirus/the-parallels-betweenspace-missions-and-covid-19-isolation.html. Accessed 2020 Jun 9.

[38] Russell A. What Submarine Crews and Astronauts Can Teach Us About Isolation. The New Yorker. 2020 Apr 9.

[39] Kelly S. I Spent a Year in Space, and I Have Tips on Isolation to Share. New York Times. 2020 Mar 21.

[40] Brown N. Retired astronaut who spent 665 days in space gives advice on pandemic isolation: Remember “bigger purpose”. CBS News. 2020 Mar 22.

[41] Tachibana K, Tachibana S, Inoue N. From outer space to Earth—The social significance of isolated and confined environment research in human space exploration. Acta Astronaut. 2017;140:273–83.

[42] Simpson AT, Doarn CR, Garber SJ. Interagency cooperation in the twilight of the Great Society: Telemedicine, NASA, and the Papago Nation. J Policy Hist. 2020;32(1):25–51.

[43] Schrage M, Kiron D. Leading with next-generation key performance indicators. MIT Sloan Management Review. 2018 Jun 16.

[44] Harvard Business Review Analytical Services. Taking the pulse of health care transformation. Harvard Business Review. 2017 Nov 22.

[45] Saxena S, Nolen W, Spencer B, Koslow L. Health care consumerism is real— and providers need to adapt. Boston Consulting Group. 2016 Aug 10.

[46] Cordina J, Kumar R, Martin CP, Jones EP. Healthcare consumerism 2018: An update on the journey. Available from: https://www.mckinsey.com/industries/healthcare-systems-and-services/ourinsights/healthcare-consumerism-2018. Accessed 2020 Jun 9.

[47] Reddy P, Onitskansky E, Singhal S, Velamoor S. Why the evolving healthcare services and technology market matters. Available from: https://www.mckinsey.com/industries/healthcare-systems-and-services/ourinsights/why-the-evolving-healthcare-services-and-technology-marketmatters. Accessed 2020 Jun 9.

[48] Mazzucato M, Roy V. Rethinking value in health innovation: from mystifications towards prescriptions. J Econ Policy Reform. 2019;22(2):101–19.

[49] Huckman R. What will U.S. health care look like after the pandemic? Available from: https://hbr.org/2020/04/what-will-u-s-health-care-look-like-after-thepandemic. Accessed 2020 Apr 7.

[50] University of Massachusetts Amherst. Center for personalized health monitoring. Available from: https://www.umass.edu/cphm/. Accessed 2019 Dec 14.

[51] University of Massachusetts Amherst. Institute for applied life sciences: Center for Human Health and Performance. Available from: https://www.umass.edu/ials/ch2p. Accessed 2019 Dec 14.

[52] Park G, Lu E, Lester M, Pye J, Sullivan R, Riccio G. Technology survey and assessment for the U.S. Army’s Objective Force Warrior Program. In: the 8th Annual International Conference on Industrial Engineering, Theory, Applications, and Practice; 2003 Nov 10–12; Las Vegas, USA.

[53] Riccio G. A case study in expeditionary community development. Unpublished work. 2020.

[54] Riccio G. Situated collaborative problem solving. Unpublished work. 2015.

[55] Zwart S, Mullenax C, Bloomberg J, Crucian B, Lee S, Ott M, Spaceflight Standard Measures. Available from: https://ntrs.nasa.gov/search.jsp?R=20180002943. Accessed 2020 Jun 9. JSC-EDAA-TN55761.

[56] Roma PG, Landon LB, Schneiderman JS. Overview of NASA Behavioral Health Performance Standard Measures. Presented at the annual meeting of the Aerospace Medical Association; 2019 May 5–9; Las Vegas, USA.

[57] Krihak M, Middour C, Gurram M, Wolfe S, Marker N, Winther S, Medical Data Architecture (MDA) Project Status. In: 2018 NASA Human Research Program Investigatorsʼ Workshop; 2018 Jan 22–25; Galveston, USA.

[58] Riccio G, Shelhamer M, Menon J. Synergy between developers and stakeholders for autonomous medical decision making. In: 2020 NASA Human Research Program Investigatorsʼ Workshop; 2020 Jan 27–30; Galveston, USA.

[59] Hafner M, Stepanek M, Taylor J, Troxel WM, van Stolk C. Why sleep matters— the economic costs of insufficient sleep: a cross-country comparative analysis. Rand Health Q. 2017;6(4):11. 10.7249/RR1791 PMC5627640 28983434

[60] Vincent C, Amalberti R. Safety strategies for care in the home. In: Safer Healthcare. Cham (Switzerland): Springer; 2016. p. 93–111. 29465922

[61] Cavuoto L, Megahed F. Understanding fatigue and the implications for worker safety. In: ASSE Professional Development Conference and Exposition; 2016 Jun 26–29; Atlanta, USA.

[62] Richardson KM. Managing employee stress and wellness in the new millennium. J Occup Health Psychol. 2017;22(3):423–8. 28150995 10.1037/ocp0000066

[63] Null C, Adduru V, Amman O, Cardoza C, Stewart M, Avrekh I, Human Performance Contributions to Safety in Commercial Aviation. Available from: https://ntrs.nasa.gov/search.jsp?R=20190001429. Accessed 2020 Jun 9. NASA/TM-2019-220254 (NESC-RP-18-01304).

[64] Reason J. The human contribution: unsafe acts, accidents and heroic recoveries. Boca Raton (US): CRC Press; 2017.

[65] National Aeronautics and Space Administration. Lifetime Surveillance of Astronaut Health (LSAH). Available from: https://lsda.jsc.nasa.gov/LSAH/LSAH_Home. Accessed 2019 Nov 27.

[66] Zierer J, Menni C, Kastenmüller G, Spector TD. Integration of ‘omics’ data in aging research: from biomarkers to systems biology. Aging Cell. 2015;14(6):933–44. 26331998 10.1111/acel.12386 PMC4693464

[67] Paschkewitz J. Simplifying complexity in scientific discovery (SIMPLEX). Available from: http://www.darpa.mil/program/simplifying-complexity-inscientific-discovery. Accessed 2020 Jun 9.

[68] Cross R, Rebele R, Grant A. Collaborative overload Available from: https://hbr.org/2016/01/collaborative-overload. Accessed 2020 Jun 9.

[69] Cross R, Davenport TH, Gray P. Collaborate Smarter, Not Harder. MIT Sloan Manag Rev. 2019;61(1):20–8.

[70] Myers J, Garcia Y, Griffin D, Arellano J, Boley L, Goodenow D, The integrated medical model: Outcomes from independent review. Available from: https://ntrs.nasa.gov/search.jsp?R=20170004392. Accessed 2020 Jun 9.

[71] National Institute of Biomedical Imaging and Bioengineering. Interagency Modeling and Analysis Group (IMAG) Available from: https://www.nibib.nih.gov/research-funding/interagency-modeling-andanalysis-group-imag. Accessed 2019 Nov 27.

[72] Leinweber L, McIntyre L, Goodenow D, Gilkey K, Myers J. A medical extensible dynamic probabilistic risk assessment tool (MEDPRAT) prototype. In: 2019 NASA Human Research Program Investigatorsʼ Workshop; 2019 Jan 22–25; Galveston, USA.

[73] Rasmussen J, Suedung I. Proactive risk management in a dynamic society. Karlstad (Sweden): Swedish Rescue Services Agency; 2000.

[74] Cohen M, Junge M. Space Station Crew Safety: Human Factors Model. In: Proceedings of the Human Factors and Ergonomics Society 28th Annual Meeting; 1984 Oct 22–26; Orlando, USA. Santa Monica (CA, US): Human Factors and Ergonomics Society; 1984. p. 908–12.

[75] Cohen M, Haeuplik-Meusburger S. What do we give up and Leave behind? In: 45th International Conference on Environmental Systems; 2015 Jul 12–16; Washington, US. ICES-2015–56.

[76] Davenport TH. Can we solve Alʼs ʻtrust problemʼ? MIT Sloan Manag Rev. 2019;60(2):18–9.

[77] Wilson HJ, Daugherty PR. Collaborative intelligence: humans and AI are joining forces. Harvard Bus Rev. 2018;96(4):114–23.

[78] Jung JJ. Computational collective intelligence with big data: Challenges and opportunities. Future Gener Comput Sys. 2017;66,87–8.

[79] Snyder J, Schultz L, Walbert T. The role of tumor board conferences in neurooncology: a nationwide provider survey. J Neuro-Oncol. 2017;133(1):1–7. doi: 10.1007/s11060-017-2416-x 28421461

[80] Knepper TC, Bell GC, Hicks JK, Padron E, Teer JK, Vo TV, Key lessons learned from Moffitt’s molecular tumor board: The Clinical Genomics Action Committee Experience. Oncologist. 2017;22(2):144–51. 28179575 10.1634/theoncologist.2016-0195 PMC5330702

[81] Chiu C. Real-time metagenomic next-generation analysis for diagnosis of infectious diseases. In: Omics Workshop; 2017 Apr 11; Houston, USA.

[82] Riccio G. Omics planning in the context of outcomes-based innovation and integration in NASA’s Human Health and Performance Directorate. Houston (TX, US): KBRwyle; 2016. Contractor report: NASA Contract NNJ15HK11B.

[83] Flores M, Glusman G, Brogaard K, Price ND, Hood L. P4 medicine: how systems medicine will transform the healthcare sector and society. Pers Med. 2013;10(6):565–76. 10.2217/PME.13.57 PMC4204402 25342952

[84] Vogt H, Hofmann B, Getz L. The new holism: P4 systems medicine and the medicalization of health and life itself. Medicine, Health Care Philos. 2016;19(2):307–23. 10.1007/s11019-016-9683-8 PMC4880637 26821201

[85] National Aeronautics and Space Administration. Space Flight Human System Standard—Volume 1: Crew Health. Available from: https://standards.nasa.gov/standard/nasa/nasa-std-3001-vol-1. Accessed 2020 Jun 9. NASA-STD-3001.

[86] National Aeronautics and Space Administration. NASA Space Flight Human System Standard—Volume 2: Human Factors, Habitability, and Environmental Health. Available from: https://standards.nasa.gov/standard/nasa/nasa-std-3001-vol-2. Accessed 2020 Jun 9. NASA-STD-3001.

[87] National Aeronautics and Space Administration. Human-rating requirements for space systems. Washington (DC, US): NASA; 2017. NPR 8705.2C.

[88] National Aeronautics and Space Administration. NASA Systems Engineering Processes and Requirements. (2020). NPR 7123.1C. Available from: https://nodis3.gsfc.nasa.gov/displayDir.cfm?t=NPR&c=7123&s=1B. Accessed 2020 Jun 28.

[89] Kusumoto Y, Makita H, Nagai K, Yamane T. Recognition of roles of various professionals by home-visiting specialists. J Phys Ther Sci. 2018;30(6):800–3. 29950767 10.1589/jpts.30.800 PMC6016306

[90] Romagnoli KM, Handler SM, Hochheiser H. Home care: more than just a visiting nurse. BMJ Qual Saf. 2013;22(12):972–4. 10.1136/bmjqs-2013-002339 PMC4120108 23940375

[91] Hanney SR, Castle-Clarke S, Grant J, Guthrie S, Henshall C, Mestre-Ferrandiz J, How long does biomedical research take? Studying the time taken between biomedical and health research and its translation into products, policy, and practice. Health Res Policy Sys. 2015;13(1):1. 10.1186/1478-4505-13-1 PMC4297458 25552353

[92] Morris ZS, Wooding S, Grant J. The answer is 17 years, what is the question: understanding time lags in translational research. J R Soc Med. 2011;104(12):510–20. 22179294 10.1258/jrsm.2011.110180 PMC3241518

[93] Ildstad ST, Evans CH Jr. Editors. Small clinical trials: issues and challenges Washington D.C. (US): National Academies Press; 2001. 25057552

[94] Kravitz R, Duan N, Eslick I, Gabler NB, Kaplan HC. Design and Implementation of N-of-1 Trials: a user’s guide. Rockville (US): Agency for healthcare research and quality, US Department of Health and Human Services; 2014.

[95] Metaphor SB, Nicholas SBD. Personalized Medicine: Time for One-Person Trials. Nature. 2015;520(7549):609–11. doi: 10.1038/520609a 25925459

[96] Tate RL, Perdices M, Rosenkoetter U, McDonald S, Togher L, Shadish W, The Single-Case Reporting guideline In BEhavioural interventions (SCRIBE) 2016: Explanation and elaboration. Arch Sci Psychol. 2016;4:10–31. 10.1037/arc0000027

[97] Sampson M. NHLBI marks year 70 of Iconic Framingham Heart Study. Available from: https://nihrecord.nih.gov/2018/02/23/nhlbi-marks-year-70iconic-framingham-heart-study. Accessed 2020 Jun 9.

[98] Framingham Heart Study (no date). Epidemiological background and design: The Framingham Heart Study. Available from: https://www.framinghamheartstudy.org/fhs-about/history/epidemiologicalbackground/. Accessed 2019 Dec 19.

[99] Fitts MA, Kerstman E, Butler DJ, Walton ME, Minard CG, Saile LG, The Integrated Medical Model: Statistical Forecasting of Risks to Crew Health and Mission Success. (2008). Available from: https://ntrs.nasa.gov/archive/nasa/casi.ntrs.nasa.gov/20080010658.pdf. Accessed 2020 Jun 28.

[100] Insider Business. Future demand for elderly care services like assisted living in-home care are rife for digital disruption. Available from: https://www.businessinsider.in/science/news/future-demand-for-elderlycare-services-like-assisted-living-in-home-care-are-rife-for-digitaldisruption/articleshow/71599033.cms. Accessed 2019 Nov 22.

[101] Hodes R. NIAʼs 2020 vision: Continued advancements in aging research! Available from: https://www.nia.nih.gov/research/blog/2020/01/nias-2020vision-continued-advancements-aging-research. Accessed 2020 Jun 9.

